# TGF-β induced TMEPAI/PMEPA1 inhibits canonical Smad signaling through R-Smad sequestration and promotes non-canonical PI3K/Akt signaling by reducing PTEN in triple negative breast cancer

**DOI:** 10.18632/genesandcancer.30

**Published:** 2014-09

**Authors:** Prajjal K. Singha, Srilakshmi Pandeswara, Hui Geng, Rongpei Lan, Manjeri A. Venkatachalam, Pothana Saikumar

**Affiliations:** ^1^ From the Department of Pathology, UT Health Science Center at San Antonio, TX; ^2^ Department of Med/Hematology & Med Oncology, UT Health Science Center at San Antonio, TX

**Keywords:** TGF-β, TMEPAI, PMEPA1, Triple Negative Breast Cancer (TNBC), Smad, PTEN

## Abstract

TMEPAI (transmembrane prostate androgen-induced) is amplified at genomic, transcript and protein levels in triple-negative breast cancers and promotes TGF-β dependent growth, motility and invasion. Tumor promotion by TMEPAI depends on two different but related actions on TGF-β signaling. Firstly, TMEPAI binds and sequesters regulatory Smads2/3 and thereby decreases growth suppressive signaling by TGF-β. Secondly, increased expression of TMEPAI decreases PTEN (phosphatase and tensin homolog) abundance, and thereby increases TGF-β dependent tumor promotive PI3K/Akt signaling. These actions of TMEPAI give rise to increased cell proliferation and motility. Moreover, signaling alterations produced by high TMEPAI were associated with oncogenic Snail expression and lung metastases. Finally, an inverse correlation between TMEPAI and PTEN levels was confirmed in triple negative breast cancer tumor samples. Together, our findings suggest that TMEPAI has dually critical roles to promote TGF-β dependent cancer cell growth and metastasis. Thus, redirected TGF-β signaling through TMEPAI may play a pivotal role in TGF-β mediated tumor promotion.

## INTRODUCTION

Triple negative breast cancers (TNBC), which lack the expression of the estrogen receptor (ER), progesterone receptor (PR), and human epidermal growth factor receptor 2 (HER2), account for more breast cancer-related deaths even though they represent only 15-25 % of total breast cancers. Therefore, understanding the functionally significant molecular alterations that occur during the pathogenesis of TNBC is essential in identifying therapeutic targets for TNBC.

Activation of PI3K/Akt signaling, which promotes cancer formation and growth, is achieved through a variety of mechanisms that includes inactivation of Phosphatase and Tensin homolog deleted on chromosome 10 (PTEN), a lipid phosphatase that converts phosphatidylinositol-3,4,5-trisphosphate to phosphatidylinositol-4,5-bisphosphate that antagonizes PI3K/Akt signaling. PTEN is one of the most frequently mutated genes in human cancer. In contrast, PTEN mutations are relatively uncommon (<5%) and PTEN protein loss is more common (~30%) in all breast cancers [1, 2]. In fact, the loss of PTEN is seen even at higher rates in ~50% triple negative breast cancers [3] and ~66% basal-like breast cancers [4]. Therefore, the negative immunohistochemical staining of the PTEN gene product associated with no deletion at the 10q23 locus suggests that there are alternate mechanisms for PTEN dysregulation other than deletion and those alternate mechanisms might play important roles in the progression of triple negative breast tumors.

In addition to PI3K/Akt signaling pathway, TGF-β pathway is also commonly deregulated leading to inactivation of its tumor suppressor activity. Tumor suppression by TGF-β is abrogated in several late stage cancers that become TGF-β dependent for growth and/or metastasis [5, 6]. Previously, we suggested that TGF-β dependent growth of aggressive breast cancer subsets depends on increased expression of a TGF-β responsive gene TMEPAI [7]. The chromosome locus 20q13 for TMEPAI/PMEPA1 is amplified in many solid tumors [8-12]. TMEPAI is overexpressed in diverse cancers [13-17] and our pioneer observation demonstrated that TMEPAI is indeed required for TGF-β dependent growth and invasive behavior of cancer cells [7]. TGF-β signals through Smad-dependent and non-canonical pathways involving mitogen activated protein kinases (MAPKs) and phosphatidylinositol 3-kinase (PI3K). Non-canonical signaling can be Smad-independent, but often needs Smad-dependent inputs[18]. In this regard, it remains unknown how normally tumor suppressive Smad signaling[19] becomes altered to promote growth and motility of triple negative cancers with amplified TMEPAI. Mechanistically, it is logical to speculate that TMEPAI might interact with R-Smads to sequester them and prevent activation by TGF-β receptors [20] in TNBC. However, the studies on molecular mechanism by which TMEPAI promotes tumor growth of TNBC were never undertaken.

Here we report that tumor promotion by TGF-β in triple negative cancer cells involves not only TMEPAI dependent abrogation of growth suppressive Smad signaling as predicted by TMEPAI-Smad interactions, but also powerful and unsuspected TMEPAI mediated activation of non-canonical signaling through PTEN (phosphatase and tensin homolog) loss and Akt activation. Amplified TMEPAI mediated not only increased proliferation but also enhanced induction of Snail by TGF-β, favoring metastasis *in vivo*. The cooperative actions of TMEPAI to decrease growth suppressive Smad signaling together with TMEPAI mediated effects on PTEN-Akt to promote growth and metastasis constitute a signaling pathology fundamentally altered from the normal state that may explain the underlying reason for TGF-β mediated tumor promotion in a large number of triple-negative breast cancers.

## RESULTS

### Prognostic significance of TMEPAI expression in metastatic breast cancer patients and its dependence on TGF-β mediated Smad signaling

**Fig. 1:**
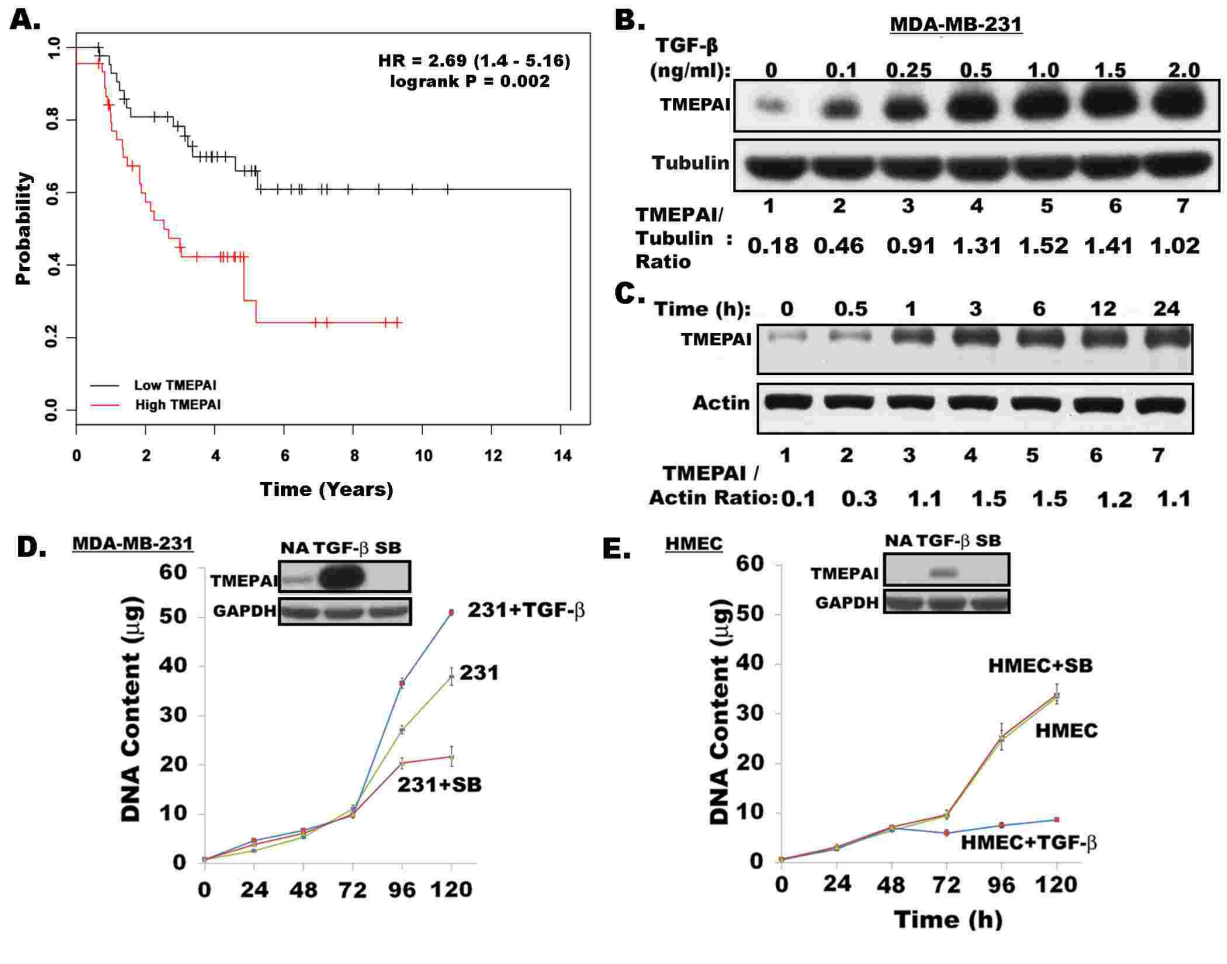
Prognostic significance of TMEPAI expression in metastatic breast cancer patients and its TGF-β dependent induction in breast cancer cells

While estrogen negatively regulates TGF-β signaling to promote growth in ER positive breast cancers[21], the highly aggressive phenotypically homogeneous triple negative breast cancers (TNBC), lacking estrogen/progesterone receptors (ER/PR) and HER2, depend on TGF-β for growth and metastasis. Previously, we showed [7] the role of TMEPAI gene product in promoting TGF-β mediated growth of TNBC cells. Here we determined the prognostic significance of TMEPAI expression in metastatic breast cancer patients by performing an initial analysis on a publicly accessible database and an online tool [22] with gene expression data and survival information (GEO:Affymetrix HGU133A and HGU133+2 microarrays). The recurrence-free survival of ER/PR negative and lymph node positive patients classified according to TMEPAI expression, at median cut-off, indicated that high TMEPAI expression correlates with shorter survival with a hazard ratio (HR) of 2.69 and a log-rank P value of 0.002 (Fig.1A) and therefore carries an unfavorable prognostic significance in ER negative and lymph node positive breast cancer patients. However, there is no significant correlation existed with TMEPAI in ER positive and lymph node positive patients (Supplemental Fig.1).

A) Kaplan-Meier curves representing the probability of recurrence-free survival of ER negative and Lymph Node positive breast cancer patients using a publicly accessible database and an online tool [22]. B) Dose dependent expression of TMEPAI protein in MDA-MB-231 triple negative breast cancer cells after 24h treatment with different doses of TGF-β. C) Time dependent expression of TMEPAI protein after treating MDA-MB-231 cells with 0.1 ng/ml of TGF-β. Growth curves of MDA-MB-231 cells (D) and HMEC (E) cells in the absence or presence of TGF-β (2 ng/ml) and SB431542 (2μM), a TGF-β RI kinase inhibitor. Inset shows relative expression of TMEPAI protein.

**Fig. 2:**
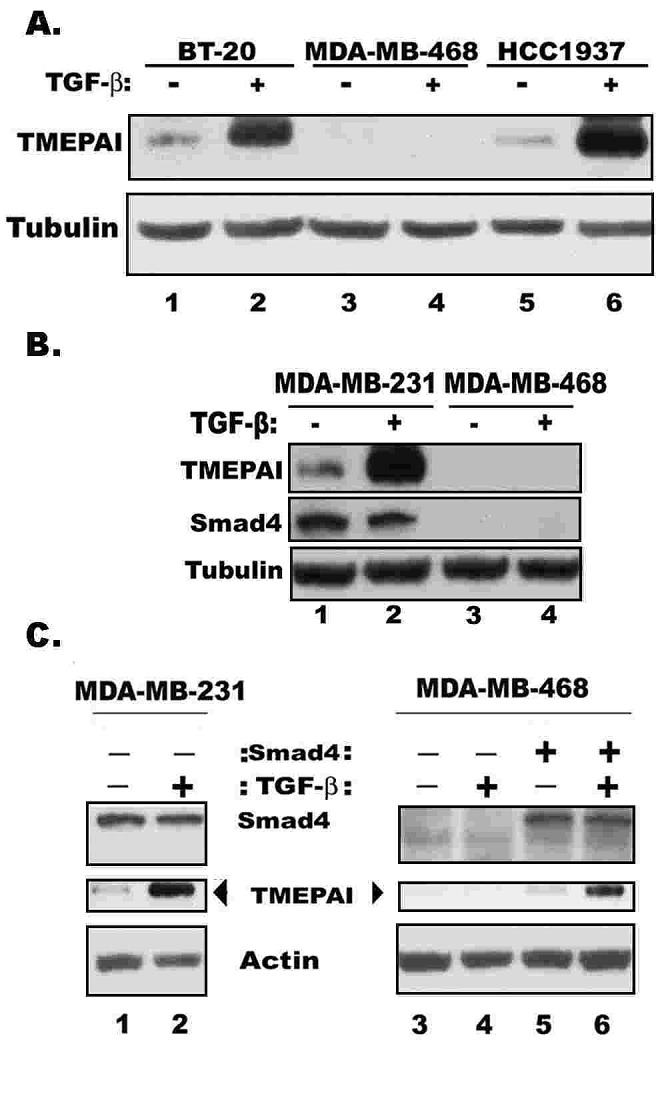
Smad requirement in TMEPAI expression A) TMEPAI expression in three different triple negative breast cancer cell lines without or with TGF-β for 24h. B) Relative expressions of TMEPAI and Smad4 in MDA-MB-231 and MDA-MB-468 cells. C) Exogenous expression of Smad4 using a retroviral vector in MDA-MB-468 cells restores TMEPAI expression and TGF-β mediated induction of TMEPAI in MDA-MB-468 cells.

TMEPAI protein in MDA-MB-231 triple negative breast cancer cells is induced by TGF-β in a concentration dependent manner as determined by exposing cells to graded amounts of TGF-β (0.1 0.25, 0.5, 1, 1.5 and 2 ng per ml) for 24 h. The induction was seen even at 100 pg/ml of TGF-β (2.5 fold) and reached maximum (8.4 fold) at 1 ng/ ml of TGFβ concentration (Fig.1B). The TMEPAI protein induction appeared to be rapid that occurred within 30 min and reached peak levels after 3 h of exposure to 1 ng/ml TGF-β (Fig.1C). Induction of TMEPAI was blocked by both transcriptional and translational inhibitors such as actinomycin D and cycloheximide, respectively (Supplemental Fig.2A). While MDA-MB-231 cancer cells are resistant to TGF-β mediated growth inhibition (Fig.1D), normal HMEC cells are sensitive (Fig.1E) to such inhibition. In some cancer cells like MDA-MB-231, TGF-β may even further stimulate their growth (see Fig.1D). Inclusion of TGF-β signaling inhibitor SB431542 in the growth medium blocked the proliferation of MDA-MB-231 cancer cells without any effect on the HMEC growth suggesting that MDA-MB-231 cells are dependent on autocrine TGF-β signaling for their proliferation but not HMEC (see Figs. 1D & 1E). Correspondingly, basal levels of TMEPAI were detected in MDA-MB-231 cancer cells, but not in normal mammary epithelial cells, HMEC (Figs.1D &1E). Importantly, TMEPAI induction by TGF-β was rather marginal in HMEC, while abundant in MDA-MB-231 cells [see Figs. 1D & 1E, insets]. TGF-β receptor I kinase inhibitor SB431542, inhibited both basal and TGF-β induced expression of TMEPAI protein (Figs.1D &1E, insets) and mRNA in MDA-MB-231 cells (Supplemental Fig. 2B), indicating positive regulation of TMEPAI expression by TGF-β. Moreover, TGF-β is much stronger than other growth factors such as EGF (epidermal growth factor) or growth factor like lipid LPA (lysophasphatidic acid) in inducing TMEPAI (Supplemental Fig.2C) suggesting the specificity for TGF-β. In addition, TMEPAI was similarly induced by TGF-β in other TNBC cell lines BT-20 and HCC1937, except in MDA-MB-468 cells (Fig.2A), because these cells lacked Smad4 expression required for TGF-β signaling (Fig.2B). In fact, exogenous expression of Smad4 in MDA-MB-468 cells restored induction of TMEPAI by TGF-β (Fig.2C) further confirming that TGF-β signaling is required for TMEPAI induction.

### TMEPAI negatively regulates Smad signaling

**Fig. 3:**
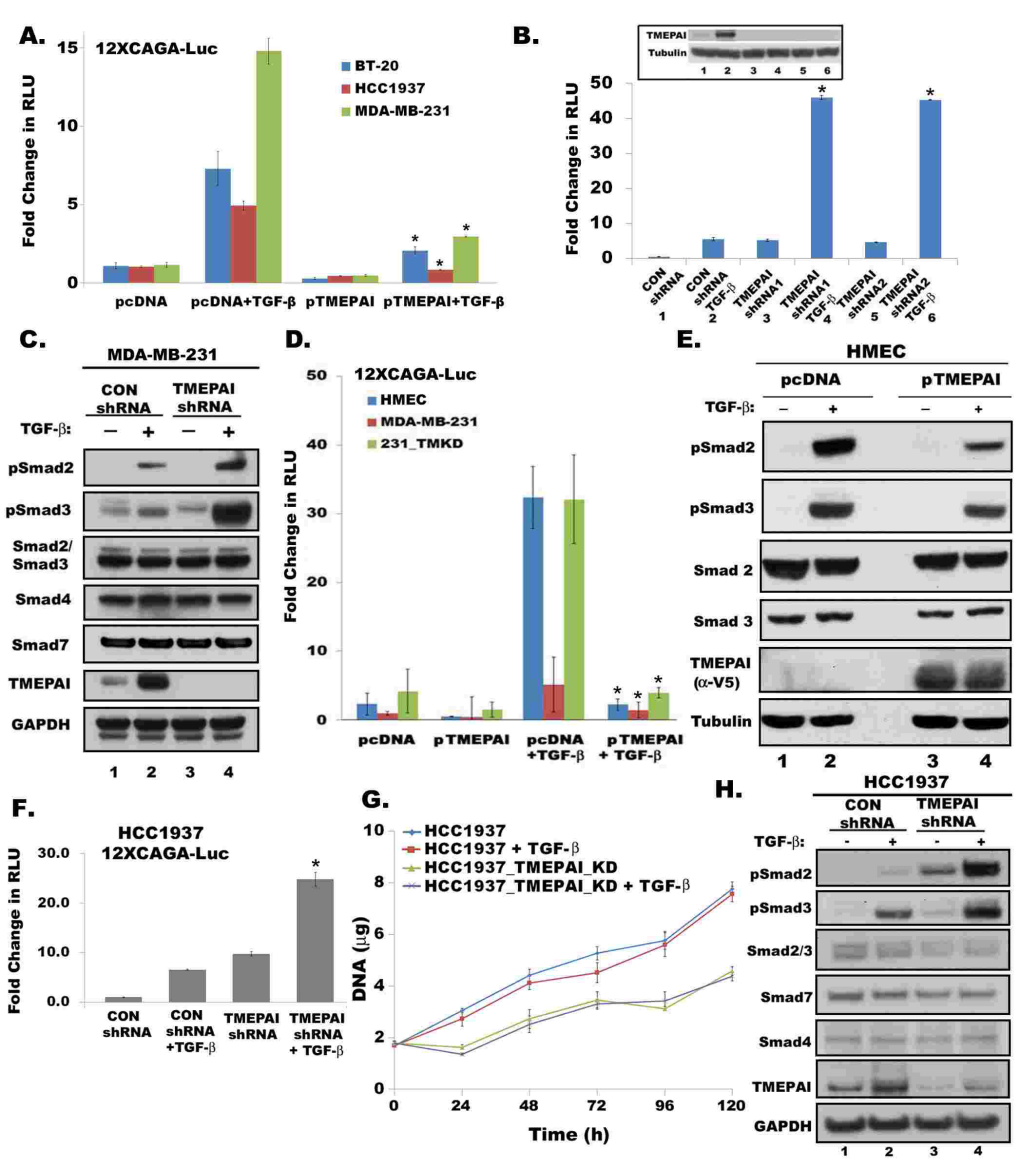
TMEPAI downregulates canonical Smad signaling All Cells were treated with or without TGF-β (2 ng/ml) for 16h. Relative light units (RLU) of Firefly Luciferase activity were normalized with Renilla luciferase activity. Data presented as mean ± SD from three independent experiments. A) Fold change in normalized 12xCAGA-Luc reporter activity in BT-20, MDA-MB-231 and HCC1937 breast cancer cells that were transfected with pcDNA or TMEPAI expression vectors. B) Effect of two different TMEPAI shRNAs (ShRNA1 and shRNA2) on Smad signaling in MDA-MB-231 cells measured as described above. Inset shows relative expression of TMEPAI by immunoblotting. C) Relative expression of phosphoSmad2/3, total Smad2/3, Smad4, Smad7 and TMEPAI and GAPDH proteins in MDA-MB-231 cells expressing control or TMEPAI shRNA. D) 12xCAGA-Luc reporter activity in HMEC and breast cancer cell lines expressing control shRNA (MDA-MB-231) or TMEPAI shRNA (231-TMKD). Mouse TMEPAI was expressed in 231-TMKD cells. E) Relative expression of phosphoSmad2/3, total Smad2/3, in HMEC expressing control pcDNA or pTMEPAI. Exogenous TMEPAI is detected by V5 antibody. F) 12xCAGA-Luc reporter activity in HCC1937 cells expressing control shRNA or TMEPAI shRNA. G) Growth curves of HCC1937 cells expressing control shRNA (HCC1937) and TMEPAI shRNA (HCC1937_TMEPAI_KD). H) Relative expression of phosphoSmad2/3, total Smad2/3, Smad4, Smad7 and TMEPAI in HCC1937 cells expressing control or TMEPAI shRNA. Asterisks denote p< 0.001.

To explore the direct effect of TMEPAI on Smad signaling, we transfected TMEPAI to increase its expression in three triple negative breast cancer cell lines and measured 12xCAGA driven luciferase (Luc) reporter activity. TMEPAI overexpression caused severe reduction in Smad signaling in all three cell lines both in the absence or presence of TGF-β (Fig.3A). To further confirm its negative effect on Smad signaling, TMEPAI expression in MDA-MB-231 cells was reduced by gene knockdown using two different shRNAs, both of which dramatically enhanced both basal and TGF-β responsive 12xCAGA-Luc activity (Fig.3B) and increased R-Smad phosphorylation without altering the levels of total Smad2/3, Smad4 and Smad7 protein levels (Fig. 3C). Because TMEPAI can sequester R-Smads [20] to diminish canonical TGF-β signaling, we tested the effects of exogenously expressed TMEPAI in HMEC (Fig. 3E). TGF-β generally elicited robust Smad signaling responses in HMEC: C-terminal phosphorylation of Smads2/3 (Fig.3E) and increased 12xCAGA-Luc activity (Fig. 3D), compared to MDA-MB-231 cells (Fig. 3B& 3C). Expression of TMEPAI in HMEC (that have no detectable basal TMEPAI) suppressed the TGF-β induced phosphorylation of Smads2/3 (Fig. 3E) and decreased 12xCAGA-Luc activity (Fig. 3D). In fact, TMEPAI expression in HMEC partially reversed the growth inhibition caused by TGF-β (Supplemental Fig. 3). Canonical signaling responses to TGF-β in MDA-MB-231 cells with control shRNA were weak as assessed by C-terminal phosphorylation of Smads2/3 or 12xCAGA-Luc activity (Figs. 3B, & 3D). On the other hand, the increased 12XCAGA-Luc activities brought about by TMEPAI knockdown in MDA- MB-231 cells were reduced by expressing mouse-TMEPAI in these cells (Fig.3D). Thus, MDA-MB-231 cells have defective Smad signaling related to pathologically high TMEPAI and the defect can be corrected by targeting TMEPAI, which resulted in reduced growth [7] of breast cancer cells. TMEPAI knockdown in another TGF-β resistant triple negative breast cancer cell line HCC1937 also resulted in increased Smad3 driven 12XCAGA signaling (Fig.3F), reduced growth of cells (Fig.3G) with enhanced R-Smad phosphorylation (Fig.3H) in the presence or absence of TGF-β just like in MDA-MB-231 cells (Fig. 3C and 3D).

**Fig. 4:**
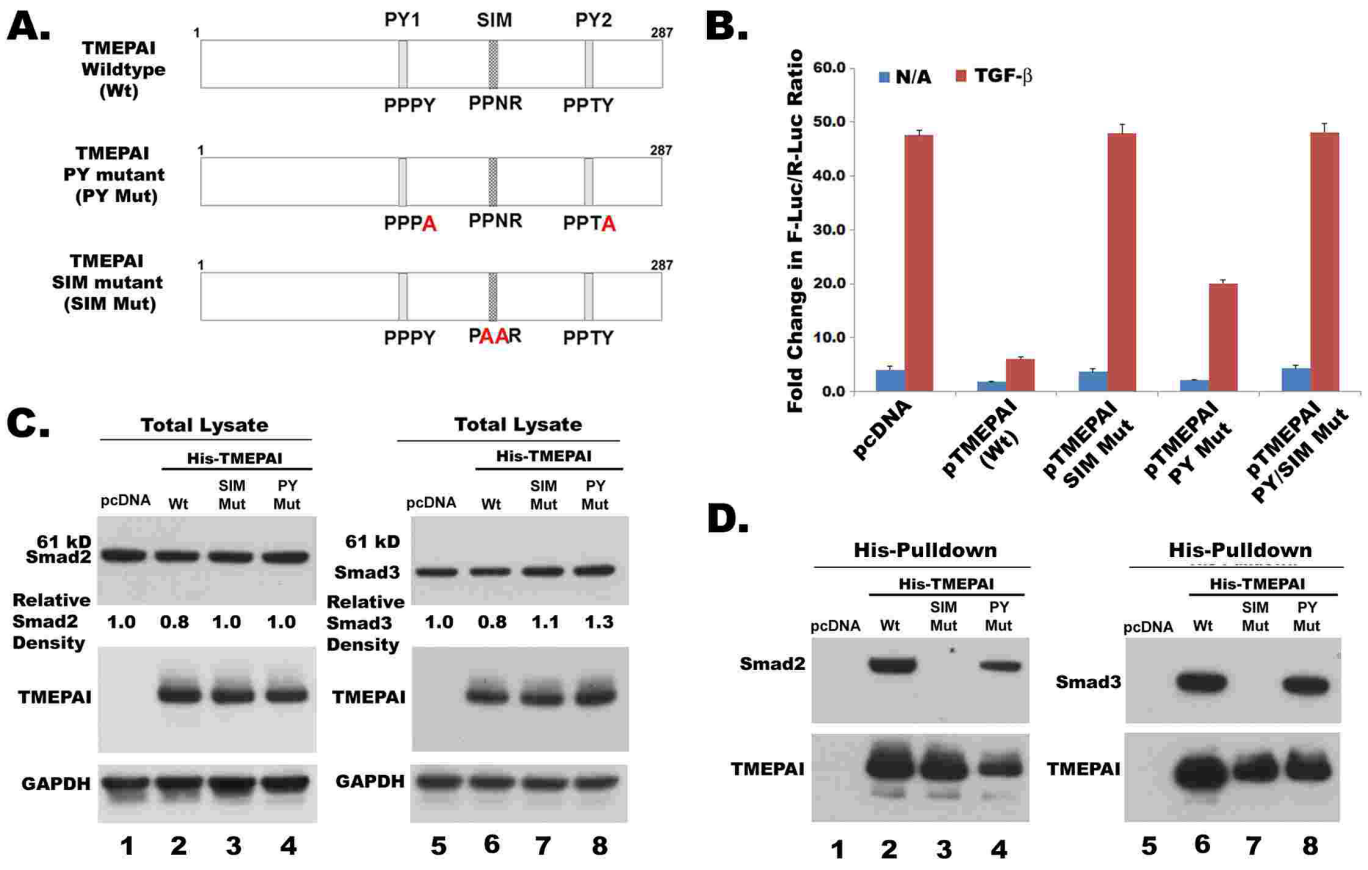
TMEPAI sequesters R-Smads A. Schematic representation of point mutations in SIM and PY motifs of human TMEPAI. SIM mutant (186PPNR189 → PAAR) and PY mutant (158PPPY161 → PPPA and 229PPTY232 → PPTA) were created by site-directed mutagenesis. B. Relative luciferase activity from MDA-MB-231 cells transiently transfected with 12X CAGA-Luc reporter and expression plasmids of TMEPAI and its mutants (See Materials section for details). Cells were treated without or with TGF-β (2ng/ml) for 16 h. C. MDA-MB-231 cells were transfected with pcDNA or pHis-TMEPAI (wild type and PY or SIM mutants) along with Flag-tagged Smad2 or Smad3 expression vectors. 24 hours later, cells lysates were collected and analyzed by Western blotting. D. Proteins from above lysates were captured by Co2+- chelate matrix and then analyzed for TMEPAI and R-Smads.

To confirm TMEPAI sequestration of R-Smads is essential for its anti-TGF-β activity as reported by Watanabe et al [20], we generated SIM (Smad interacting motif) and PY (NEDD4 WW domain interacting motif) mutants in TMEPAI (Fig.4A). While wild type TMEPAI completely suppressed Smad dependent 12XCAGA reporter activity, SIM mutant has no effect (Fig.4B). However, it was surprising that the PY mutant, which abolished the interaction between TMEPAI and NEDD4 [17], inhibited 12XCAGA reporter activity only partially instead of completely (Fig.4B). We speculated that PY mutations could have altered the conformation of TMEPAI such that interaction of SIM domain with R- Smads may be affected. Expression of Smad2 and Smad3 proteins were lower in cells that were transfected with wild type TMEPAI vector than in pcDNA or TMEPAI mutant vectors (Fig.4C). Both R-Smads were pulled down by wild type TMEPAI and PY mutant but not SIM mutant (Fig.4D). However, PY mutant was slightly less efficient than wild type TMEPAI in pulling down R-Smads (Fig.4D), which is consistent with our speculation above.

### TMEPAI promotes TGF-β mediated non-canonical PI3K/Akt signaling required for cancer cell growth

**Fig. 5:**
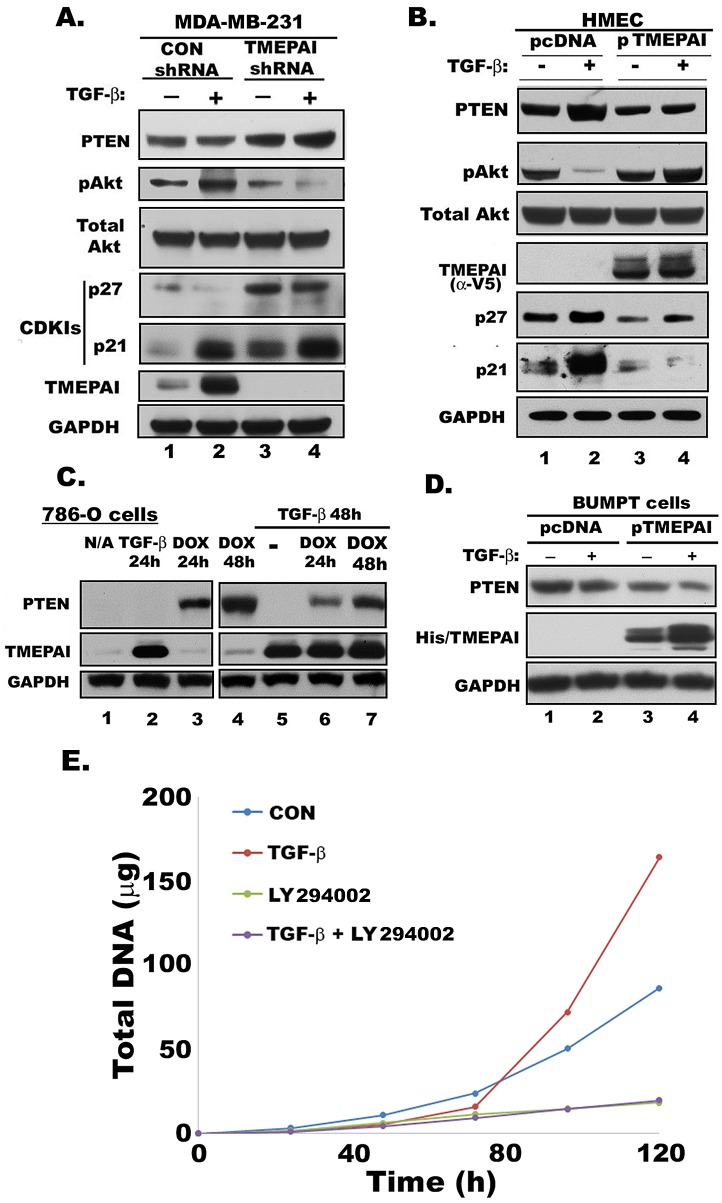
Effect of TMEPAI on PTEN, Akt, pAkt and CDKIs expression A. Relative expression determined by Western blotting of PTEN, phosphorylated Akt (pAkt), total Akt, p21, p27, TMEPAI and GAPDH in MDA-MB-231 cells expressing control (CON shRNA) or TMEPAI shRNA without and with TGF-β (2 ng/ml) treatment. B) Western blots of HMEC expressing control vector (pcDNA) or human TMEPAI (pTMEPAI) in the absence or presence of TGF-β. C) Relative levels of PTEN and TMEPAI in PTEN null 786-O human renal carcinoma cells expressing doxycycline (DOX) inducible PTEN vector in the absence or presence of TGF-β (2 ng/ml) and/or Doxycycline (1μg/ml). D) Relative expression of PTEN protein in mouse proximal tubule cells (BUMPT) expressing pcDNA or mouse TMEPAI (pTMEPAI) without or with TGF-β (2 ng/ml). TMEPAI is detected with anti-His antibody. E) Growth curves of MDA-MB-231 cells in the absence or presence of TGF-β (2 ng/ml) and LY294002(10μM) alone or together.

It is well known that TGF-β, a pleiotropic cytokine, can function both as a growth suppressor of healthy tissues and a promoter of tumor cell growth in advanced cancers. However, the basis for this reversal in function of TGF-β also known as the TGF-β paradox is poorly understood. In addition to Smad dependent actions, TGF-β signaling also employs non-Smad effects, especially in cancer cells, such as activation of the PI3K pathway, which utilizes Akt, a proto-oncogene intermediate in the pathway[23]. PI3K/Akt signaling is often regulated by the tumor suppressor PTEN, which antagonizes this signaling pathway by dephosphorylating the PI3K product phosphatidylinositol 3,4,5- trisphosphate [24]. We noted earlier that tumors from TMEPAI knockdown MDA-MB-231 cells had elevated PTEN levels [7]. Here we show that in MDA-MB-231 triple negative breast cancer cells basal levels of PTEN are enhanced by TMEPAI knockdown, which further increased by TGF-β treatment (Fig. 5A). Correspondingly, Akt phosphorylation was high in response to TGF-β in cancer cells that express control shRNA but lower in TMEPAI knockdown cells, which had higher PTEN levels (Fig. 5A).

Because activity and expression of the cdk-inhibitors and growth suppressors p21 and p27 are controlled by PTEN and Akt [25, 26], breast cancer cells with control shRNA (low PTEN, high phospho-Akt) had low levels of p21 and p27 whereas cells with TMEPAI knockdown (high PTEN, low phospho-Akt) had increased p21 and p27 basally as well as with TGF-β (Fig. 5A and 5B). Complementary observations were made in HMEC, where PTEN levels were normally high in HMEC and were increased further by the addition of TGF-β (Fig. 5B). The TGF-β induced increase of PTEN in HMEC was accompanied by suppressed phosphorylation of Akt (Fig. 5B) and increased expression of Cdk-inhibitors, p21 and p27 (Fig. 5B). On the other hand, HMEC with TMEPAI overexpression showed low PTEN, high Akt phosphorylation and correspondingly decreased p21 and p27 expression both basally and in presence of TGF-β (Fig. 5B).

Since, PTEN can be regulated transcriptionally as well as by degradation [27], to determine whether TGF-β effects on PTEN downregulation in normal mammary epithelial cells may be mediated by TMEPAI, first we examined the effects of TGF-β in a system where transcriptional effects native to cells cannot affect PTEN levels. For this purpose, we used PTEN null 786-0 carcinoma cells expressing the reverse transcriptional activator rtTA that drives a heptameric tetO sequence with minimal CMV promoter to express PTEN only in the presence of tetracycline or its analogs[28]. Our experiments showed that PTEN was strongly induced when doxycycline was included in the medium; however, if TGF-β was present in addition to doxycycline, TMEPAI was induced as expected, but PTEN levels were substantially lower (Fig. 5C, compare lanes 3, 4 and lanes 6, 7), suggesting that TGF-β treatment might cause loss of the doxycycline induced PTEN. To further confirm that induced TMEPAI is responsible for decreased PTEN levels, we exogenously expressed TMEPAI in mouse proximal tubule cells (BUMPT) that express higher levels of PTEN compared to cancer cells. As shown in Fig. 5D, exogenous expression of TMEPAI resulted in decreased PTEN (compare lanes 1 and 3 in Fig. 5D). TGF-β treatment, which resulted in further increase of TMEPAI protein, further reduced PTEN levels (Fig.5D, compare lanes 3 & 4). Unlike HMEC, which showed increased PTEN in the presence of TGF-beta (Fig. 5B) with growth inhibition (Fig. 1E), wild type BUMPT cells showed decreased PTEN in the presence of TGF-beta (Fig.5D), which is in total correspondence with their abnormal growth in the presence of TGF-beta (data not shown). To address whether TMEPAI-PTEN-PI3K/Akt axis is necessary for tumor progression, we tested the effect of PI3K inhibitor LY294002 on the MDA-MB-231 cancer cell growth. As shown in Fig. 5E, LY294002 completely inhibited breast cancer cell growth both in the absence or presence of TGF-β suggesting that PI3K/Akt pathway is essential for cancer cell growth.

### TMEPAI promotes PTEN Protein degradation

**Fig. 6:**
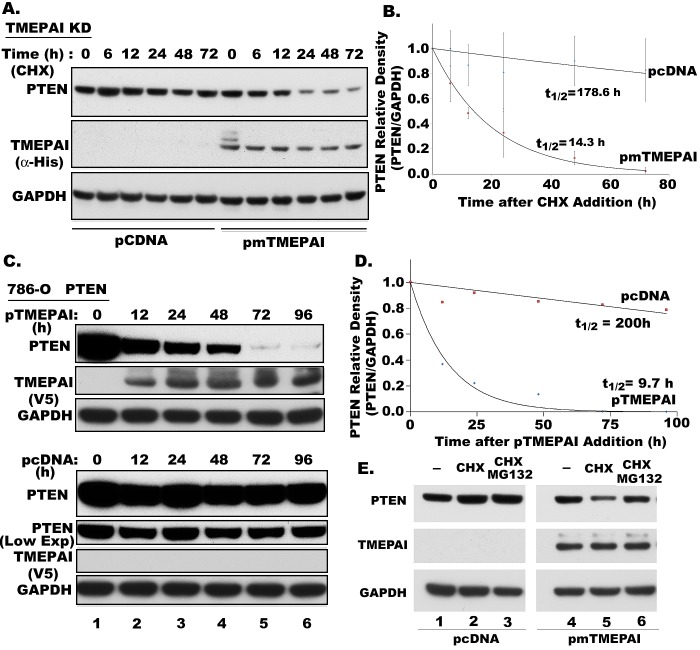
Effect of TMEPAI on PTEN protein turnover A) Time course of PTEN in cycloheximide (CHX; 10μM) treated TMEPAI knockdown MDA-MB-231 cells (TMEPAI KD) that were transfected with pcDNA or mouseTMEPAI (pmTMEPAI) vector. Cell lysates were analyzed for PTEN, His tagged- TMEPAI and GAPDH by Western blotting. B) Relative density of PTEN was plotted against time after treatment with CHX from three independent experiments. PTEN half-life (t1/2) was calculated from decay rates in PTEN levels. C) Time course of PTEN after induction by doxycycline (DOX) in PTEN null 786-O human renal carcinoma cells following the removal of DOX and expression of pcDNA or pTMEPAI vectors. D) The half-life of PTEN was calculated as above in 786-O cells transfected with pcDNA and human TMEPAI. The results are an average from two independent experiments. E) PTEN and TMEPAI levels in TMEPAI knockdown MDA-MB-231 cells expressing empty vector (pCDNA3.1) and mouse TMEPAI and treated with CHX (10μM ) and a protease inhibitor MG132 (10μM) for 12h.

**Fig. 7:**
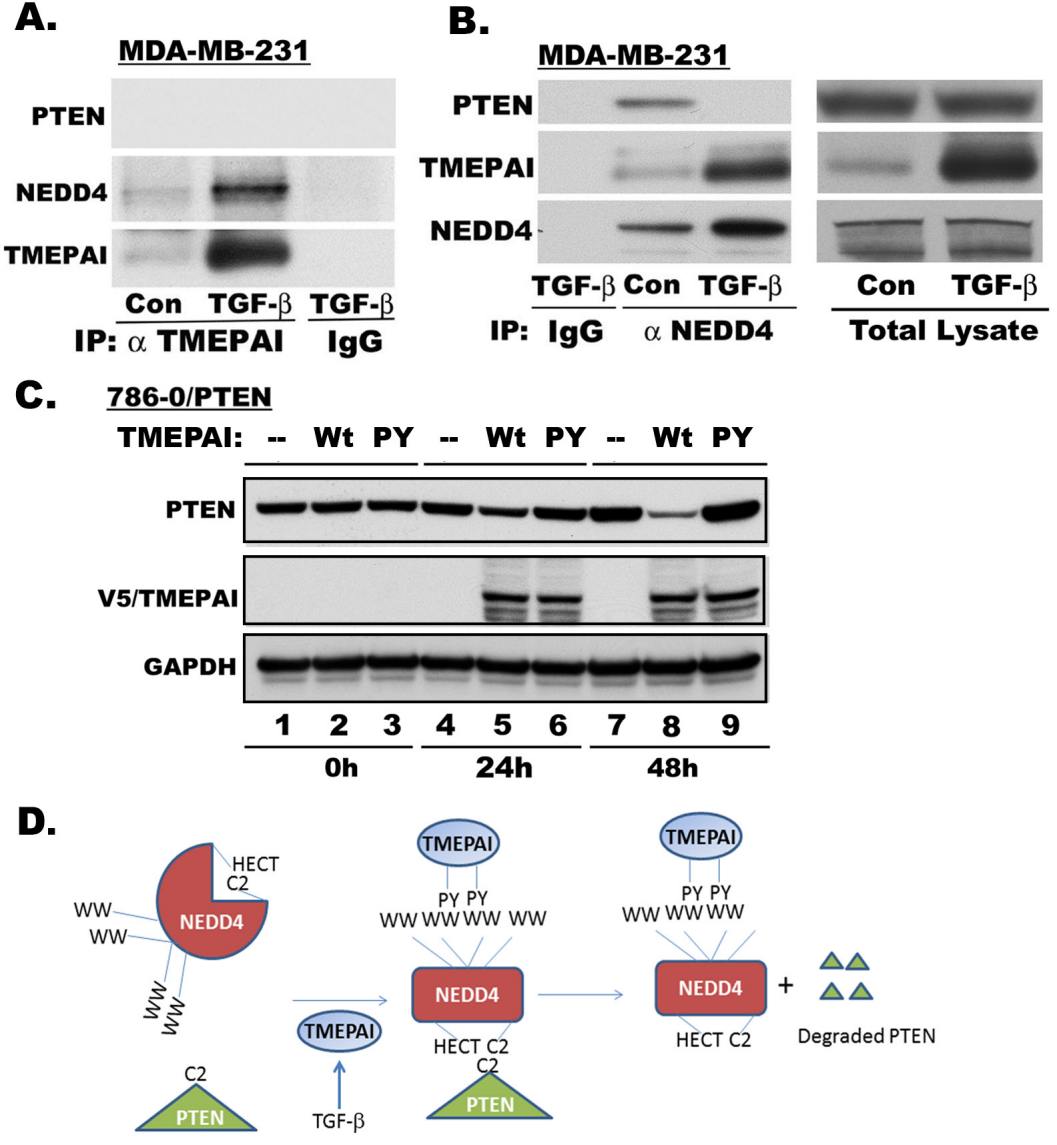
Downregulation of PTEN levels by TMEPAI A) Immunoprecipitation by α-TMEPAI mouse monoclonal antibody of MDA-MB-231 cell lysates treated without or with TGF-β and analyzed for TMEPAI, NEDD4 and PTEN by western blotting. Mouse IgG was used as control. B) Immunoprecipitation by α--NEDD4 rabbit polyclonal antibody of MDA-MB-231 cell lysates treated without or with TGF-β and analyzed for TMEPAI, NEDD4 and PTEN by western blotting. Rabbit IgG was used as control. C). Following PTEN induction by DOX (1μg/ml) in 786-O cells for 24 h, DOX was removed and cells were transfected with pcDNA, wild type (wt) or PY mutant of TMEPAI expression vectors. Cells were harvested at 0, 24h and 48h time points and analyzed for PTEN, V5-tag (TMEPAI) and GAPDH by Western blotting. D). Mechanistic model of enhanced PTEN turnover by TMEPAI and NEDD4 in cancer cells.

Since PTEN levels are consistently increased in TMEPAI-deficient cells (Fig.5A) and decreased in TMEPAI overexpressing cells (Fig. 5B & 5D), we tested if TMEPAI promotes PTEN turnover to reduce PTEN levels. We examined endogenous PTEN protein levels to determine whether TMEPAI regulates the stability of the PTEN protein, in the presence of cycloheximide (CHX), an inhibitor of protein translation. In TMEPAI knockdown cells, the PTEN protein appeared to turnover at slower rate with a probable extrapolated half-life of ~ 179h (Fig.6A, and 6B). In contrast, mouse TMEPAI expression in these cells caused rapid turnover of PTEN with half-life decreased to ~ 14h. To further confirm this observation that decreased PTEN in the presence of TMEPAI is not due to either cytotoxic effect of CHX or through an indirect effect on transcription, we used 786-O cells that express PTEN by doxycycline inducible vector. Following the induction of PTEN, doxycycline was removed and human TMEPAI was exogenously expressed and monitored PTEN levels by Western blotting over a period of time. As shown in Fig.6C, while PTEN levels dramatically reduced in TMEPAI expressing 786-O cells, PTEN levels were relatively stable in cells harboring control vector. Similar to TMEPAI knockdown breast cancer cells, the half-life of induced PTEN in 786-O human renal carcinoma cells was also high (~200h), which was reduced to ~10 h by exogenous expression of human TMEPAI (Fig. 6D). Our results indicate that PTEN is much more stable than what was previously reported with a half-life of ~8 h for PTEN [29, 30]. However, most of those earlier turnover studies used tagged PTEN [29, 30]. In fact when we compared the stability of His-tagged PTEN with unmodified endogenous PTEN in BUMPT cells, we found chimeric PTEN with His-tag degraded rapidly with half-life of 5.4 h, while endogenous PTEN remained stable for at least 24 h after the addition of CHX (supplemental Fig.4). Decrease in PTEN by TMEPAI appears to be mediated by increased proteasomal degradation, as MG 132, a broad spectrum inhibitor of proteasomes, prevented PTEN loss (Fig. 6E, compare lanes 5 and 6) Our immunoprecipitation studies showed no direct interaction between PTEN and TMEPAI as they could not be co-precipitated by α-TMEPAI antibody (Fig. 7A) or α-PTEN antibody (not shown). However, both TMEPAI and PTEN independently interacted with NEDD4, a WW-domain containing HECT E3 ligase, as both the proteins were co-precipitated by α-NEDD4 antibody (Fig. 7B). Interestingly, TGF-β treatment caused increased association of NEDD4 with its antibody as well as TMEPAI, while PTEN disappeared from this complex presumably because of its degradation. Since PY motifs in TMEPAI are responsible for its interaction with WW domains in NEDD4 like E3 ligases [17], we tested whether this interaction is required to promote PTEN degradation. Indeed, double PY mutant of TMEPAI failed to induce PTEN degradation in 786-O cells compared to wild type TMEPAI (Fig.7C). Based on our studies, we proposed a possible model by which TGF-β induced TMEPAI may be involved in promoting PTEN turnover (Fig.7D). Overall, our results clearly indicate that TMEPAI promotes PTEN loss through increased PTEN turnover (Figs. 5 and 6), which probably enhances non-canonical PI3K/Akt activity in triple negative breast cancer cells in response to TGF-β.

### Role of TMEPAI-PTEN-PI3K/Akt axis in mediating metastatic behavior of breast cancer cells

**Fig. 8:**
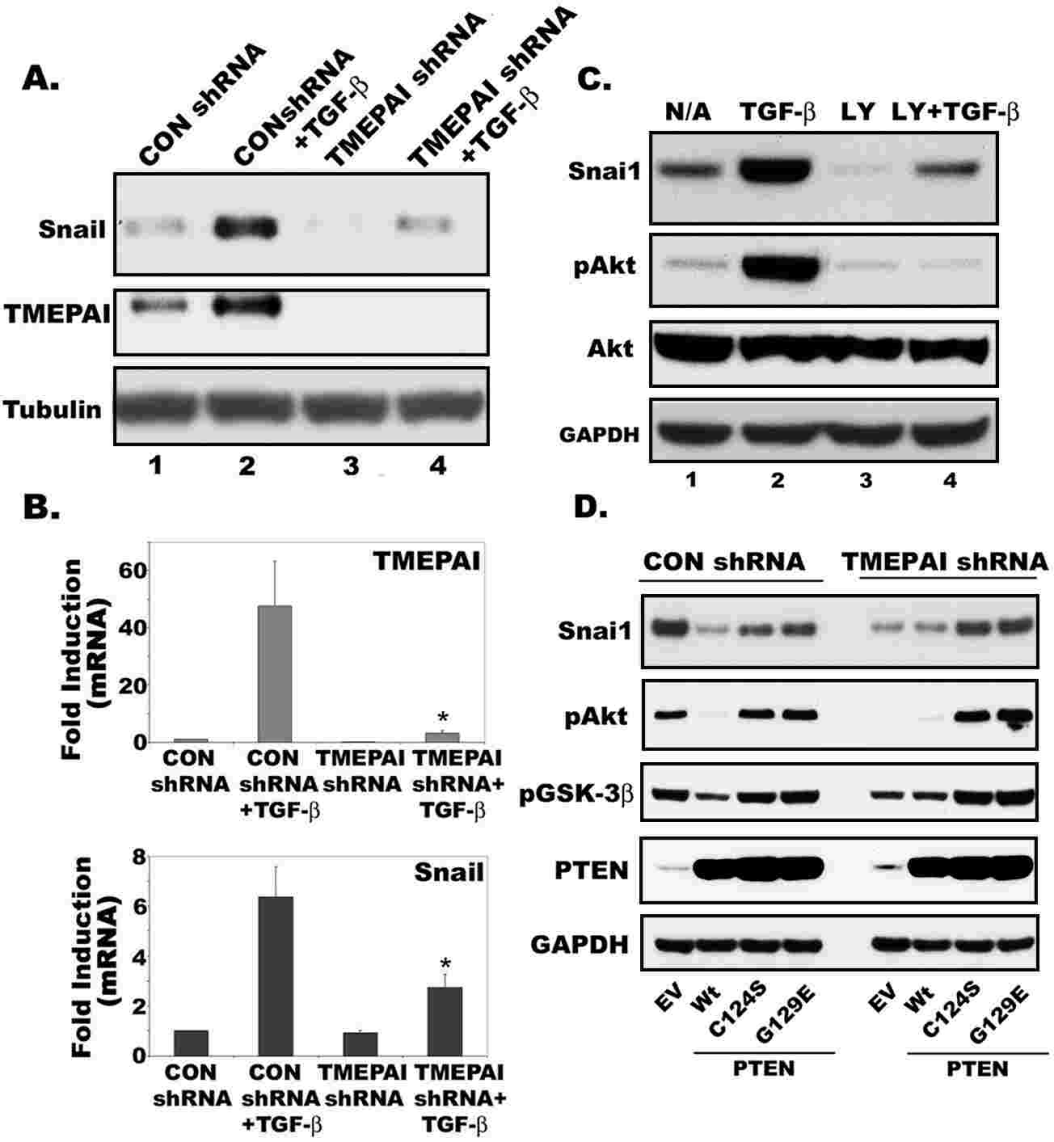
Role of TMEPAI-PTEN axis in promoting Snai1 expression in breast cancer cells Relative expression of Snail1 and TMEPAI proteins (A) and mRNAs (B) in MDA-MB-231 cells expressing control and TMEPAI shRNA without or with TGF-β (2 ng/ml). C) Effect of PI3K inhibitor, LY294002 (10μM) on Snail1 expression and Akt activation (phospho Akt; pAkt) in MDA-MB-231 cells without or with TGF-β (2 ng/ml) stimulation. D) Effect of wild type (Wt) and mutated (C124S, G129E) PTEN, expressed by Adenoviruses, on CON shRNA and TMEPAI shRNA expressing MDA-MB-231 cells for the expression of Snail1, pAkt, pGSK-3β, PTEN and GAPDH. EV: Empty virus.

We showed previously that TMEPAI knockdown in MDA-MB-231 cells markedly decreased the size of tumor xenografts compared to corresponding cells with control shRNA[7], which is consistent with an important role for TMEPAI in TGF-β dependent growth not only in culture[7] (Fig. 1D), but also *in vivo* [7]. Apart from its role in cell growth (Fig.5E) and survival, activation of the PI3K/Akt pathway is involved in cancer cell migration [31] and is linked with increased invasiveness of many tumors [32]. These effects of PI3K/Akt are antagonized by the tumor suppressor PTEN [33] that we now show to be a target for TMEPAI. Increased PI3K/Akt signaling leads to up-regulation of the transcription factor Snail [34] and reduces cell–cell contacts and promotes epithelial-to-mesenchymal transition [35]. When functional correlations between Snail and TMEPAI in MDA-MB-231 cells were investigated, we found that both Snail protein and mRNA were markedly increased by treatment of cells with TGF-β, along with the expected induction of TMEPAI. The effects of TGF-β to increase Snail were blocked by TMEPAI knockdown both at protein and mRNA level (Fig. 8A and 8B). Since Snail expression can govern tumor invasiveness [36], TMEPAI knockdown, which reduces Snail expression, decreased MDA-MB-231 cell motility and invasion properties [7]. To test whether PI3K/Akt activation is responsible for Snail induction by TGF-β in breast cancer cells, we treated cells with PI3K inhibitor LY294002. As shown in Fig.8C, LY294002 blocked TGF-β mediated Snail induction and Akt activation by TGF-β in MDA-MB-231 cells. To confirm that up regulation of PTEN activity is involved in reducing PI3K/Akt activity in TMEPAI knockdown cells, we tested the effect of dominant negative PTEN mutants in recovering Akt activation, Snail induction and cell growth of TMEPAI knockdown cells. As shown in Fig.8D, reduced pAkt and Snail in TMEPAI knockdown cells were recovered when dominant negative mutants of PTEN (C124S and G129E, lipid and protein phosphatase inactive mutant and lipid phosphatase alone inactive mutant, respectively) were expressed using adenoviral vectors. Interestingly, Slug (Snai2) another gene that governs tumor invasiveness, whose expression was also regulated by TMEPAI just like Snai1in cancer cells (data not shown).

**Fig. 9:**
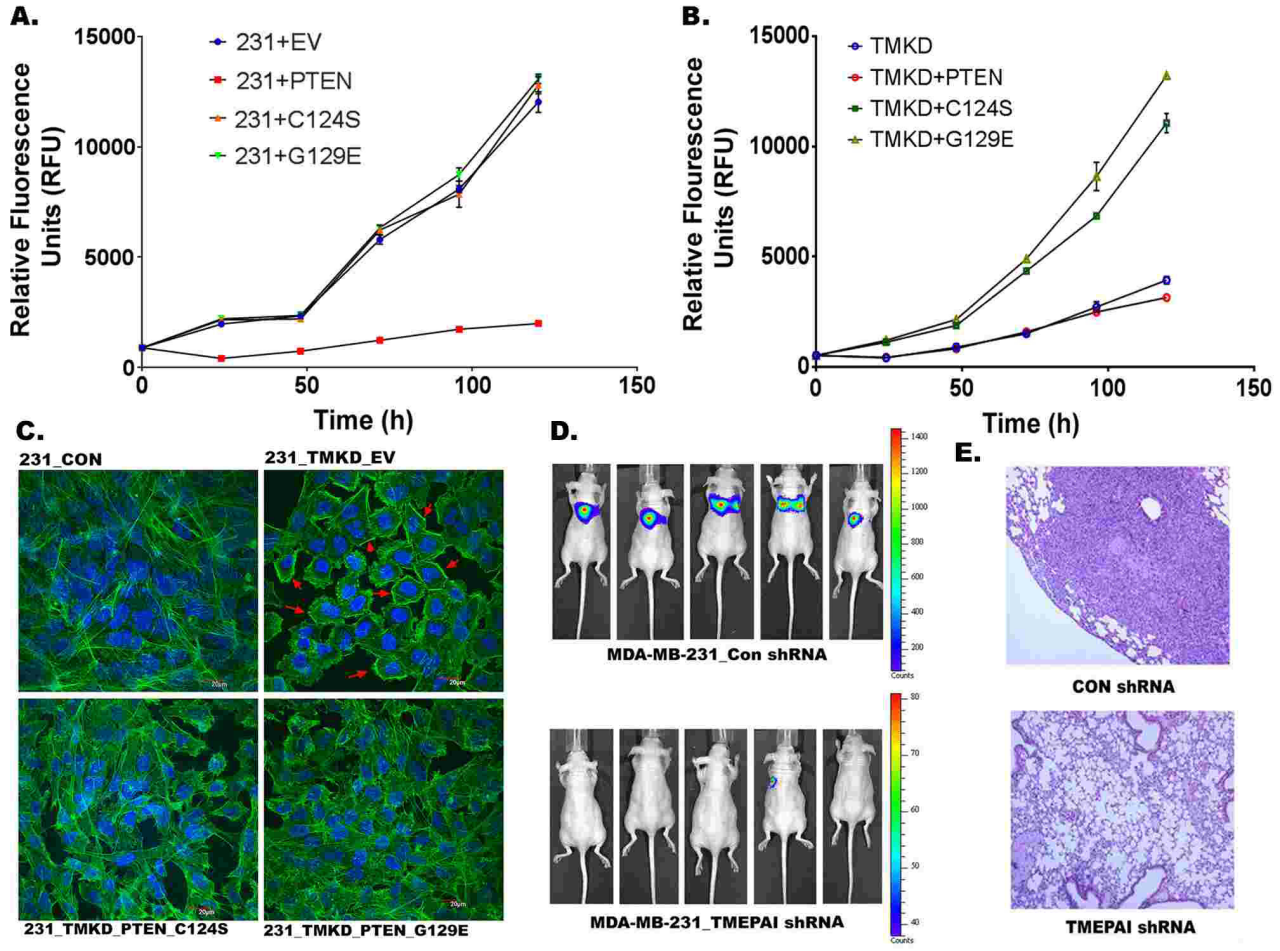
Role of TMEPAI-PTEN axis in growth and metastatic behavior of breast cancer cells A). Wild type PTEN expression in MDA-MB-231 cells (express control shRNA) inhibits their growth. B). mutant PTEN ((C124S or G129E) expression in growth inhibited TMEPAI knockdown cells (MDA-MB-231 cells expressing TMEPAI shRNA) promotes their growth. C) Mutant PTEN (C124S or G129E) expression alters the F-Actin distribution in TMEPAI knockdown cells (231-TMKD). F-actin was labeled with phalloidin (green) and nuclei were stained with DAPI (blue). Cells infected with wild type PTEN (not shown) had similar actin staining as empty virus (EV). Scale bar: 20μ. D. Representative dorsal bioluminescence images of mice taken at day 35 following intravenous injection of MDA-MB-231-GFP- Luc cells stably expressing control shRNA (CON-shRNA) or TMEPAI shRNA showing lung metastasis. E. Histology of lung alveoli from mice injected with MDA-MB-231 cells expressing control CON shRNA or TMEPAI shRNA and stained with H&E.

To find out whether TMEPAI mediated alterations in PTEN levels are responsible for altered cancer cell growth, we tested the effect of Wild type and dominant mutant PTEN on the growth of wild type and TMEPAI knockdown MDA-MB-231 cells. While normal PTEN severely inhibited the growth of wild type 231 cells (Fig.9A), it had no significant effect on the growth of TMEPAI knockdown cells (Fig.9B). In contrast, while mutant PTEN expression had no effect on wild type breast cancer cell growth (Fig.9A), they profoundly increased the growth of TMEPAI knockdown cells that are otherwise growth retarded (Fig. 9B).

To determine whether altered PTEN levels are also responsible for structural changes in the cell to promote tumor invasiveness, actin stress fibers in MDA-MB-231 cells were localized by labeling for F- actin with FITC-labeled phalloidin and analyzed by confocal microscopy. Wild type MDA-MB-231 cells expressing control shRNA (231-CON) exhibited prominent actin stress fibers (Fig.9C, top left panel), while TMEPAI shRNA expression (231-TMKD) resulted in the reorganization of the cellular actin cytoskeleton to the cortical regions of the cell body indicated with arrows (Fig.9C, top right panel). Expression of dominant negative mutants of PTEN, using adenoviral vectors, evoked strong actin reorganization back to the formation of actin stress fibers in TMEPAI knockdown cells (Fig.9C, bottom panels) clearly suggesting that altered PTEN levels under TMEPAI expression are responsible for not only Akt activation but also cell structural changes. Moreover, inhibited growth of TMEPAI knock down cells was recovered by expressing PTEN dominant mutants in these cells (Fig. 9B). Using a standard metastasis assay, we further examined the role of TMEPAI in the metastatic behavior of MDA-MB-231 cells *in vivo*. As shown in Fig.9D, TMEPAI knockdown in MDA-MB-231 cells greatly decreased tumor lung metastases *in vivo* following intravenous injection compared to corresponding cells with control shRNA (Figs. 9D and 9E).

### Correlation between TMEPAI and PTEN expression in TNBC patients by IHC

**Fig. 10:**
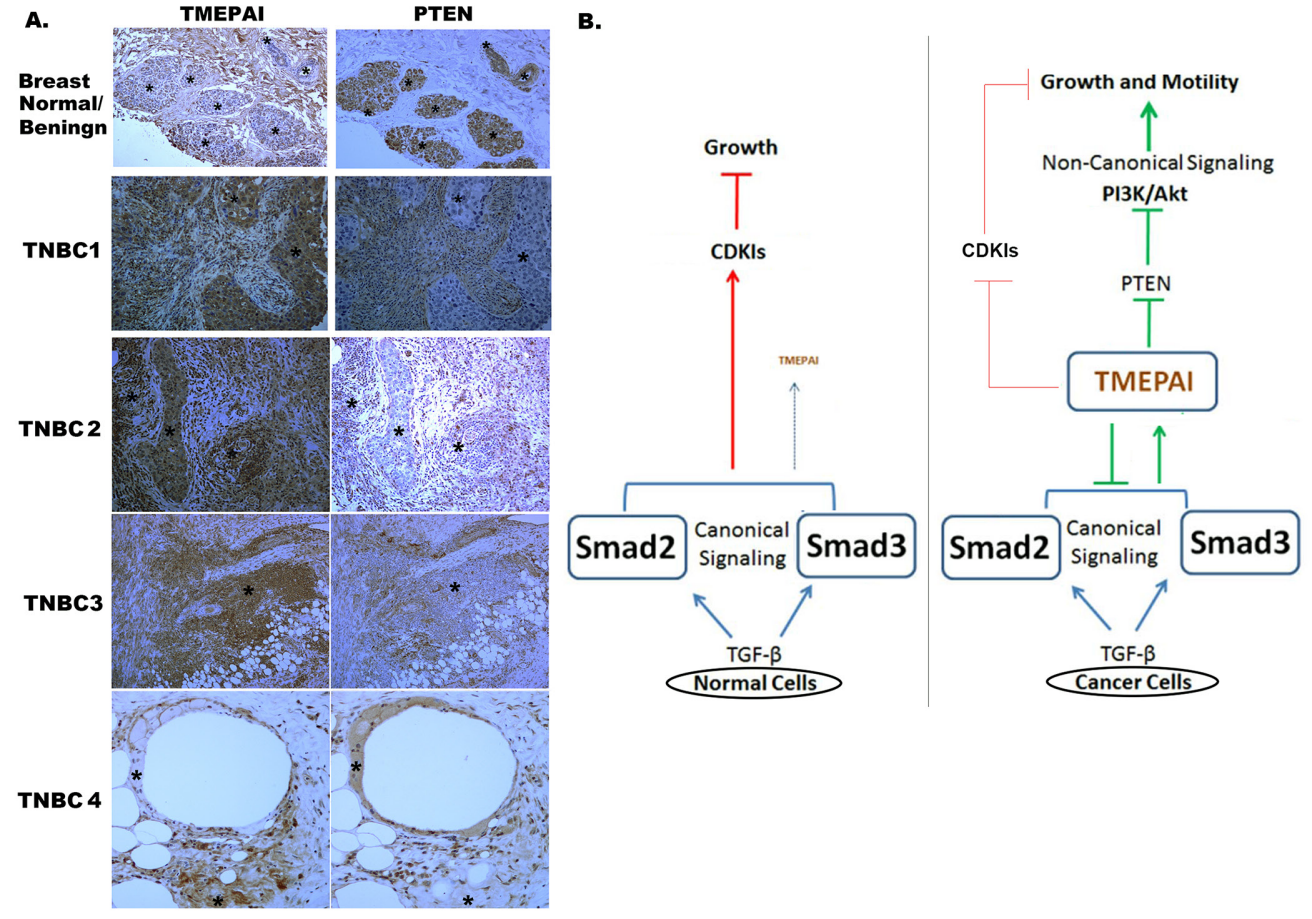
Inverse correlation between TMEPAI and PTEN expression in TNBC tumors by IHC A) Immunohistochemistry of TMEPAI and PTEN proteins in normal/ benign human breast tissue and four human triple negative breast cancer tissues (TNBC1-4). Areas represented by asterisks (*) indicate TMEPAI or PTEN positive stain is associated with low or no PTEN or TMEPAI stain respectively. B) Model showing a central role for TMEPAI in shifting the balance of canonical TGF-β signaling towards non-canonical signaling pathways in cancer cells. In cancer cells, TGF-β induced TMEPAI protein inhibits canonical TGF-β signaling by sequestering R-Smads and induces non- canonical PI3K/Akt signaling by reducing PTEN and promotes growth and metastasis of cancer cells. In normal cells TMEPAI protein expression is tightly regulated to prevent its expression by TGF-β.

Since our findings support the role of TMEPAI as a potent negative regulator of PTEN tumor suppressor, thus emerging as a potential oncogenic marker in breast cancer, we next conducted a comprehensive expression analysis of TMEPAI and PTEN in TNBC tumors and surrounding normal/benign tissues by IHC. Generally, there is an inverse correlation existed between TMEPAI and PTEN expression in both normal and breast cancer tissues. Typical staining patterns are presented in Fig.10A. Cytoplasmic PTEN expression was very strong in benign epithelial cells (Fig.10A, top right panel) but largely reduced in most breast cancerous tissues (Fig. 10A, see 4 right bottom panels). TMEPAI was usually not expressed in benign mammary ductal epithelia (Fig.10A, top left panel), but expressed in the breast cancer tissues (Fig. 10A, see 4 left bottom panels). Interestingly, the surrounding stromal tissue of normal/benign epithelium appeared to be stained for TMEPAI but not PTEN, which needs to be further explored with normal breast tissue from disease free individuals to learn the significance of this observation. Based on above results we proposed a model, in which how TMEPAI may subvert growth suppressive TGF-β signaling in breast cancer cells into growth promotion by inhibiting canonical Smad signaling through R-Smad sequestration and promoting non-canonical PI3K/Akt signaling through PTEN decrease (Fig.10B). Finally, we evaluated TMEPAI and PTEN protein expressions using immunohistochemistry on a tissue microarray, constructed from 45 TNBC specimens. We noticed a negative correlation between the expression of PTEN and TMEPAI in triple negative breast tumors analyzed by IHC (Supplemental Fig. 5). In 40/45 samples there is a significant inverse correlation existed between PTEN and TMEPAI [r (Pearson correlation coefficient) = -0.505; with P (two-tailed) = 0.004]. We found 31 samples (69%) had low PTEN and high TMEPAI, 9/45 (20%) had high PTEN and low TMEPAI. And rest (5/45:11%) lacked expression of both PTEN and TMEPAI suggesting that a large proportion of triple negative breast tumors may be dependent on the TMEPAI expression to drive tumor growth and metastasis.

## DISCUSSION

The results reported here suggest that TMEPAI appears to have a dynamic role in regulation of TGF- β canonical and non-canonical signaling and help to explain how increased TMEPAI protein subverts normally tumor suppressive TGF-β signaling to promote TGF-β dependent proliferation of triple negative breast cancer cells and enhance their metastatic propensity. In this respect, it is noteworthy that TMEPAI is expressed in several TNBC tumors (~69%). In this article we report that tumor promotion by TGF-β/TEMPAI axis is mediated by several pathways. Primarily overexpression of TMEPAI results in resistance to growth inhibition by TGF-β, through the actions of TMEPAI to sequester and decrease R- Smad activation, thereby reducing growth suppressive canonical TGF-β signaling [Fig. 3 and [20]]. Secondly, TMEPAI can activate a non-canonical TGF-β pathway by degrading PTEN by ubiquitin proteasome pathway (Figs.5, 6 and 7) and thus enhances oncogenic PI3K/Akt signaling with potential to support proliferation and metastasis.

Our results suggest that canonical growth suppressive TGF-β signaling is functionally preserved in both HCC1937 and MDA-MB-231 cells but is decreased by the actions of amplified TMEPAI. This is inferred by the effects of TMEPAI knockdown to “correct” the defect at steps involving R-Smad activation, transcription by Smads reflected by TGF-β reporter (12XCAGA-Luc) assays (Fig.3). Thus, one reason for TGF-β dependent proliferation of triple negative breast cancer cells (Fig.1) is the suppression of growth suppressive canonical TGF-β signaling by amplified TMEPAI. As predicted by the findings of Watanabe et al [20], TMEPAI was found to bind and sequester R-Smads and thereby decrease their activation. Decreased canonical TGF-β signaling caused by R-Smad sequestration was associated with impaired expression of cdk-inhibitor, p27 (Fig. 5). We suggest that decrease of such TGF-β regulated growth suppressive proteins leads to increased proliferation of the cancer cells with amplified TMEPAI. This inference is supported by the effects of TMEPAI knockdown in MDA-MB-231 cells which led to increased R-Smad activation and canonical signaling (Fig. 3), increase of p27 protein (Fig. 5) and decreased cell growth [see[7] and Fig. 3G]. Our results also suggest that TGF-β dependent proliferation of the cancer cells requires TMEPAI-PTEN-PI3K/Akt signaling axis, as PI3K inhibitors (Fig.5E) or wild type PTEN (Fig. 9A) could completely inhibit cell growth. Knockdown of TMEPAI led to increased PTEN with corresponding decrease in Akt activation and proliferation as well as increased expression of cdk-inhibitors p27 and p21. Akt generally promotes proliferation through mTOR[37] and p27 degradation [38]. With intact R-Smads in cancer cells, TGF-β induced TMEPAI would activate Akt, translocate FoxO to cytoplasm, and decrease p21 transcription. However, we predict R-Smad-TMEPAI- Akt mediated proliferation of cancer cells may depend more on the suppression of p27 than of p21, since Smad3 is a cofactor for p21 transcription [39] and Smad3 knockdown would inhibit p21 induction.

Of note, TMEPAI may be responsible for imparting oncogenic role to TGF-β because its' expression is stronger in human TNBC than normal cells when exposed to TGF-β. High levels of TMEPAI in the triple negative cancer cells were associated with low PTEN and high Akt activation. Confirmation that TMEPAI regulates PTEN and PI3K signaling was shown both by TMEPAI knockdown in cancer cells, which resulted in increased PTEN protein and decreased Akt phosphorylation (Figs. 5A) and by TMEPAI overexpression in normal cells (Fig. 5B), which caused reduced PTEN and increased Akt phosphorylation, mimicking the signaling status of the cancer cells. Although the exact mechanisms by which TMEPAI promotes increased PTEN turnover are not yet clearly established, the involvement of NEDD4-1 or other E3 ubiquitin ligases of the NEDD4 family is likely based on their interaction with TMEPAI through WW domains [17] and their earlier reported involvement in PTEN degradation [29, 40]. Overall, TMEPAI positively regulates tumorigenesis via promoting the proteasome mediated degradation of PTEN (Fig. 6) and potentiates TGF-β mediated PI3K/Akt activation in a PTEN dependent manner (Figs. 5 &9). Although current study identified NEDD4 as a binding partner for both TMEPAI and PTEN (Fig. 7B), we could not rule out other WW domain containing E3-HECT ligases [40, 41]. TMEPAI per se may not interfere with the interaction between NEDD4-1 and PTEN (Fig. 7C), because different spatially separated domains on NEDD4 are interacting with these proteins (Fig.7C) while C2 and HECT domains of NEDD4 bind to C2 domain of PTEN[42], WW domain of NEDD4 interacts with the PY motifs of TMEPAI, suggesting that TMEPAI may indirectly modulate PTEN protein stability through NEDD4 like proteins. Further studies are warranted to find differences in the binding affinity of PTEN with NEDD4 or other related E3 ligases and ubquitination patterns (mono or poly) of PTEN in the presence and absence of TMEPAI. Overall, our study revealed that TMEPAI potentiates tumorigenic role of TGF-β by inhibiting canonical signaling through R-Smad sequestration (current study and [20]) and promoting non canonical signaling through activation of PI3K/Akt signaling by reducing PTEN.

Since TMEPAI abundance can promote Akt activation that inhibits nuclear localization of FoxO proteins by phosphorylating them, this non-canonical signaling input is yet another mechanism that could account for the loss of growth arresting properties of TGF-β through Smads. Activated Akt in cancer cells can stimulate protein synthesis and consequently cell growth [43, 44] through activation of mTOR, S6 kinase and eukaryotic initiation factor 4E-binding protein 1(4E-BP1). By activating Akt, TMEPAI could also decrease the expression of cdk-inhibitor p27 by promoting its degradation through phosphorylation effects on the SKP2/p27 axis [26], thereby promoting proliferation. Moreover, in order to effect growth arrest, Smad3 and Smad4 form complexes with FoxO family of transcription factors to induce p15Ink4b and p21Cip1 [45]. Additionally, Akt activity is anti-apoptotic and in this fashion can promote proliferation. Thus, in cases where TGF-β induces apoptosis and growth inhibition [46, 47], TMEPAI has the potential to counteract not only the growth inhibitory effects of TGF-β but also its pro- apoptotic actions by activating Akt, thereby contributing to tumor promotion by TGF-β.

In addition to promoting cell proliferation, TGF-β increases the invasive and metastatic propensity of TGF-β dependent cancers[48]. Our current work has revealed a plausible basis for increased invasiveness and metastasis of triple negative breast cancer cells caused by TGF-β dependent TMEPAI mediated non canonical signaling. High levels of TMEPAI in MDA-MB-231 cells favored TGF-β dependent induction of Snail and Slug mRNA and protein, and knockdown of TMEPAI markedly blunted the expression of Snail under basal conditions as well as after TGF-β treatment (Fig. 8). While Snail induction is Smad dependent [49], its expression is also stringently controlled by degradation through GSK3β mediated phosphorylation that is in turn affected by PI3K signaling [35]. Because we found that high TMEPAI activates downstream PI3K-Akt signaling that has the potential to increase Snail expression through the signaling intermediate GSK3β, our results provide an explanation for the invasive potential of triple negative cancer cells with amplified TMEPAI. Since Snail expression has been related to tumor invasion and metastasis [36], suppression of lung metastases by TMEPAI knockdown in MDA-MB-231 cells could be accounted for by its suppressive effects on Snail (Fig.8A and 8B).

Finally, we examined the clinical relevance of TMEPAI and PTEN in human breast cancer tissues. TMEPAI expression would be predicted to decreases the levels of PTEN in human breast cancer samples. We confirmed this hypothesis by assessing the expression of TMEPAI and PTEN in the TMAs of TNBC tissue cores. There was a significant inverse correlation between TMEPAI and PTEN levels in TNBC tumors. Collectively, these results imply that the expression of TMEPAI leads to resistance to growth inhibition by TGF-β and contributes to the downregulation of PTEN in human breast cancers, which may lead to tumor progression. This conclusion was further supported by the observation that PTEN levels were high in TMEPAI negative tumors. Our findings have important implications in that TMEPAI, which contributes to the PTEN-dependent oncogenic activity of TGF-β, would be a suitable biomarker in distinguishing cancer in the early stages of development and provide clues for the development of a molecular target to treat TMEPAI positive cancers [16, 50, 51] including aggressive TNBC.

## METHODS

### Materials

Antibodies were obtained from sources indicated. TMEPAI (Abnova, CA), pSmad3, V5 epitope tag (Rockland, PA), Smad2/3 (BD Biosciences, CA), Smad 4 (SantaCruz biosciences, CA), Flag and Tubulin (Sigma, MO), GAPDH (R&D, MN), Actin and p27 (Epitomics, CA), pSmad2, Akt, pAkt, p21, phosphoGSK-3β, Snail, PTEN and Nedd4 (Cell Signaling, MA), Smad 7 (Imgenex, CA). Other reagents were obtained as indicated below. ProLong® Gold Antifade Reagent with DAPI, Fluorescein phalloidin (Lifetechnologies, NY), TGF-β (R&D, MN), LY294002 (Calbiochem, CA), H33258 (Bisbenzimide), actinomycin D, SB431542 and cycloheximide (Sigma, MO); doxycline (Clonetech, CA).

### Cell lines, Gene Expression, Western blot analysis and Knockdown

MDA-MB-231, BT-20, HCC1937 and MDA-MB-468 breast cancer cells and HMEC were maintained and grown as previously described (7) and cell lines were authenticated by genomic STR profile. Mutations in SIM domain of TMEPAI were generated using a Quick Change site-directed mutagenesis kit (Agilent/Stratagene, CA). Western blots were performed and analyzed as described before (7). TMEPAI knockdown was achieved using lentiviral vectors (shRNA1: 5′-GAGCAAAGAGAAGGATAAACA-3′, shRNA2: 5′- GTCCCTATGAATTGTACGTTT-3′) as before [7]. Control shRNA, targets non mammalian green fluorescent protein gene. Retroviral vector expressing Smad4, lentiviral vector expressing GFP- Luciferase, and expression plasmids for TMEPAI, Smad2 and Smad3 were kind gifts.

### Cell proliferation

Cell growth assays were performed by measuring total cell DNA content with Hoechst 33258 dye using a fluorescence reader [7].

### Quantitative real-time-PCR and Transfection, Luciferase assays

Quantitative RT-PCR was performed as described before for TMEPAI [7] and Snai1 (primers for Snai1 are Forward: 5′- CTTCCAGCAGCCCTACGA-3′; Reverse: 5′-AGCCTTTCCCACTGTCCTC-3′). Cells were transfected with Smad binding element driven-firefly luciferase (12XCAGA-Luc) and renilla luciferase expression vectors. Dual luciferase assays were performed according to vendor instructions (Promega, WI). Expression of human and mouse TMEPAI was achieved by transfection. All experiments were repeated at least three times.

### Adenovirus infection

Cells were infected with empty virus (EV), wild type PTEN (Wt) and PTEN mutants (C124S and G129E) at 10 moi for 48h. After 48 h, total lysates were made for Western blotting. Cells were fixed with 4% paraformaldehyde, after 48 h of infection with adenoviruses, and processed further for phalloidin staining by following vendor's protocol.

### Immunohistochemistry

Paraffin embedded slides of TNBC tumors (From Institutional breast pathology core) and breast tissue microarray slides (US Biomax Inc., Rockville, MD) were used for TMEPAI and PTEN immunohistochemistry (IHC). In brief, after, deparaffinization and rehydration, sections were used for antigen retrieval in 99°C 1 mM Tris-EDTA for 20–25 min. All the sections were washed with TBST, quenched endogenous peroxidase with 3% hydrogen peroxide for 15 min and washed with TBST. Sections were blocked with 2.5% horse serum before incubating with primary antibodies (TMEPAI and PTEN) followed by ImmPRESS HRP polymer-conjugated secondary antibodies (Vector labs, CA). All sections were developed with DAB chromogen followed by hematoxylin counterstain, dehydrated and mounted.

### Protein-Protein interactions

His-Tagged TMEPAI proteins expressed in MDA-MB-231 were purified by Co2+-Talon Metal Beads (Clontech, CA) under native conditions as recommended by the supplier. Co- purified samples were analyzed by Western blotting using appropriate primary antibodies. Immunoprecipitations were carried out as described before [52].

### Bioluminescence imaging

GFP-luciferase labeled MDA-MB-231 cells (0.1×106), expressing control or TMEPAI shRNA, were injected through lateral tail vein of anaesthetized nude mice (5 animals in each group). Tumor development in mice was monitored using bioluminescence imaging system (XenogenIVIS Spectrum, Caliper Life Sciences, MA) for 5 weeks. All experiments and procedures were done in accordance with the Institutional Animal Care and Use Committee (IACUC) guidelines.

### Statistical Analysis

Data is presented as the mean ± SD. All statistical tests were performed using GraphPad Prism version 6 for Windows (GraphPad Software, La Jolla CA USA). Statistical significance was determined using the unpaired two-tailed Student t test, with a p value < 0.05 considered significant. Pearson's correlation was used to examine associations between two variables. P values for the survival curves have been calculated using a log-rank test.

## SUPPLEMENTARY MATERIAL FIGURES


