# Supplementary material for: TGF-β induced TMEPAI/PMEPA1 inhibits canonical Smad signaling through R-Smad sequestration and promotes non-canonical PI3K/Akt signaling by reducing PTEN in triple negative breast cancer
Source: Genes Cancer. 2014 Sep;5(9-10):320–36. doi: 10.18632/genesandcancer.30 (PMC4209604; doi:10.18632/genesandcancer.30)
Supplement: Supplementary file 1 [file ganc-05-320-s001.pdf]

TMEPAI inhibits canonical smad signaling through R-Smad sequestration and promotes non-canonical PI3K/Akt signaling by reducing PTEN in triple negative breast cancer

Supplementary Material

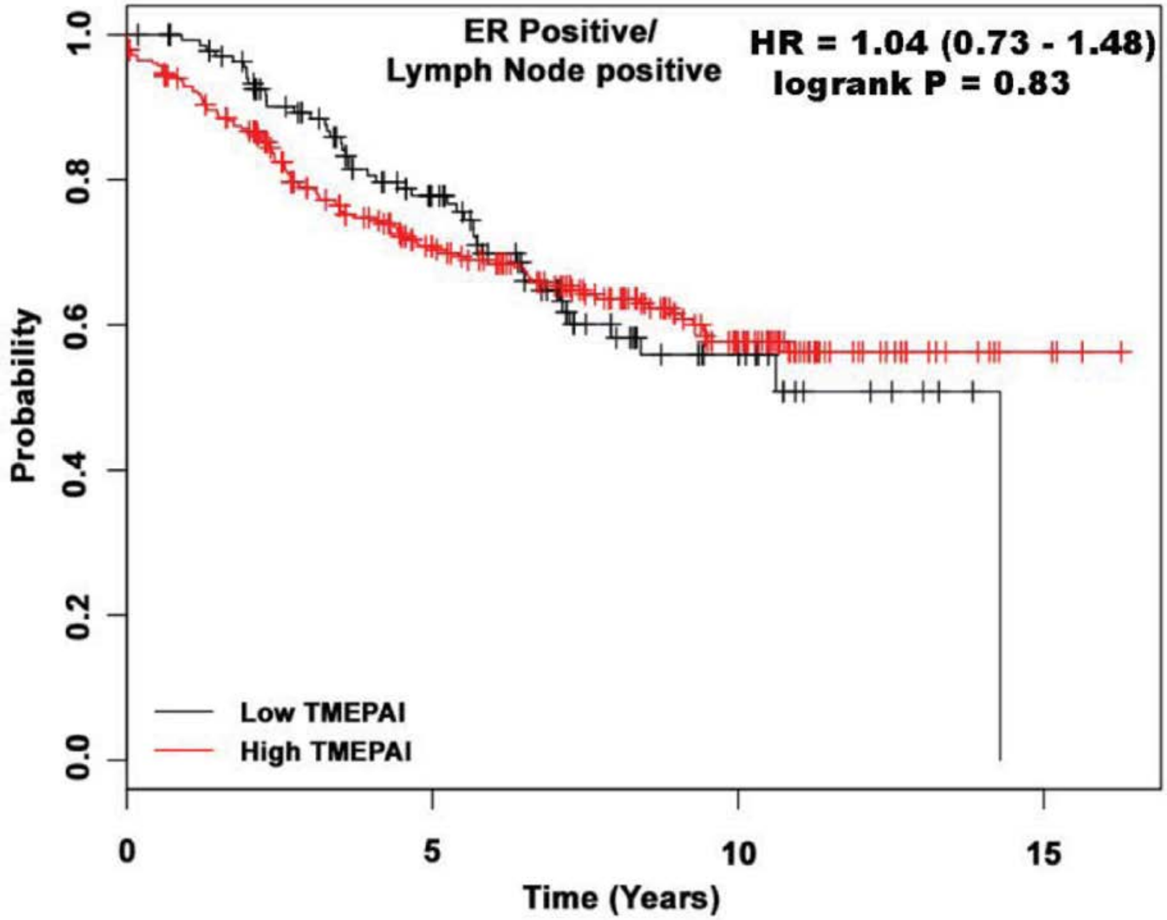

Supplemental Figure 1: Prognostic significance of TMEPAI expression on ER/PR positive and lymph node positive breast cancer patients.

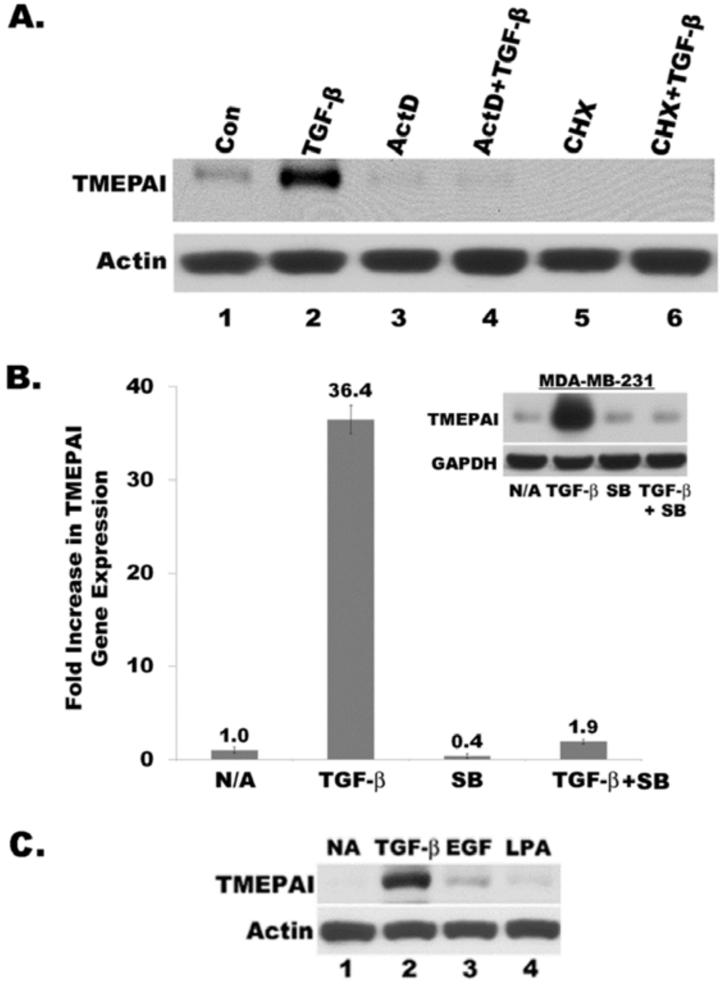

Supplemental Figure 2: Increased transcription and translation in presence of TGF-β is responsible for TMEPAI induction by TGF-β. A. Effect of transcription inhibitor, actinomycin D (Act D, 8 μM) or translational inhibitor cycloheximide (CHX, 25μM) on TMEPAI protein expression in MDA-MB-231 cells that were pretreated with above inhibitors for one hour before stimulated by TGF-β (2ng/ml) for 6h. B. Effect of TGF-β and SB on TMEPAI mRNA expression in MDA-MB-231 cells after 24h of treatment by QPCR. Inset shows relative expression of TMEPAI protein. C. Relative stimulation of TMEPAI protein in MDA-MB-231 cells by different growth factors after 24h of treatment. Transforming growth factor-β (TGF-β) stimulates maximal TMEPAI expression in cancer cells than epidermal growth factor (EGF) or lysophasphatidic acid (LPA), which stimulates GPCR signaling.

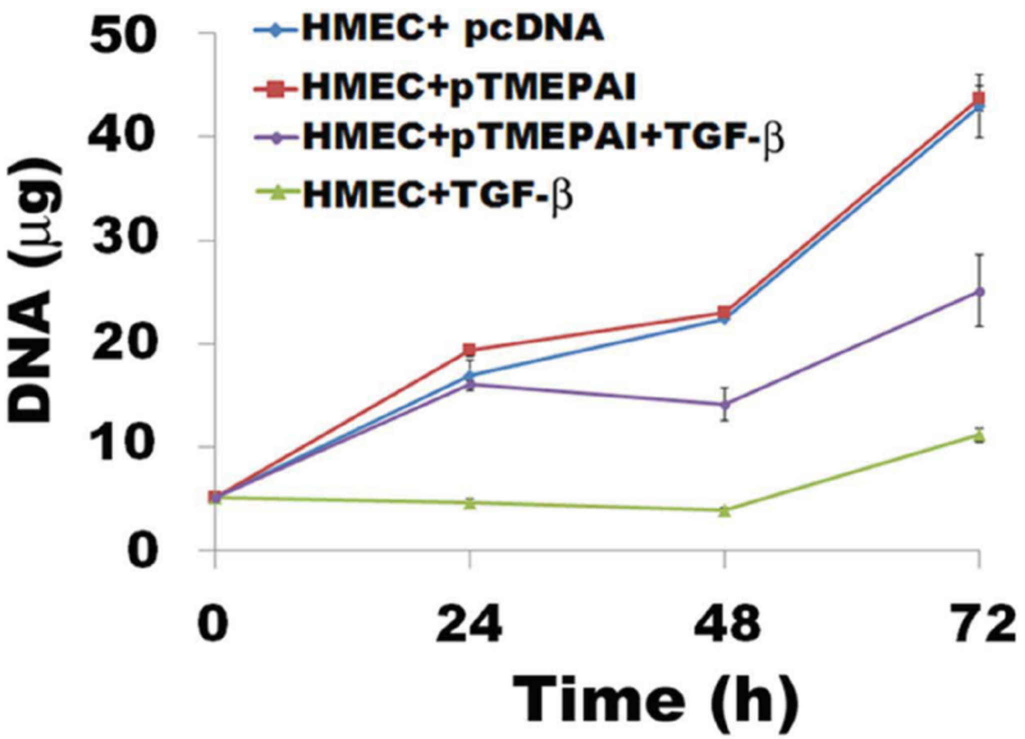

Supplemental Figure 3: TGF-β Growth inhibition of HMEC is partially reversed by TMEPAI expression. HMEC were transfected either with empty vector (pcDNA) or human TMEPAI expression vector. Following transfection, cells were treated with TGF-β (2ng/ ml) in fresh medium every 24 h. Cell proliferation assays were performed by measuring DNA content as described before (7).

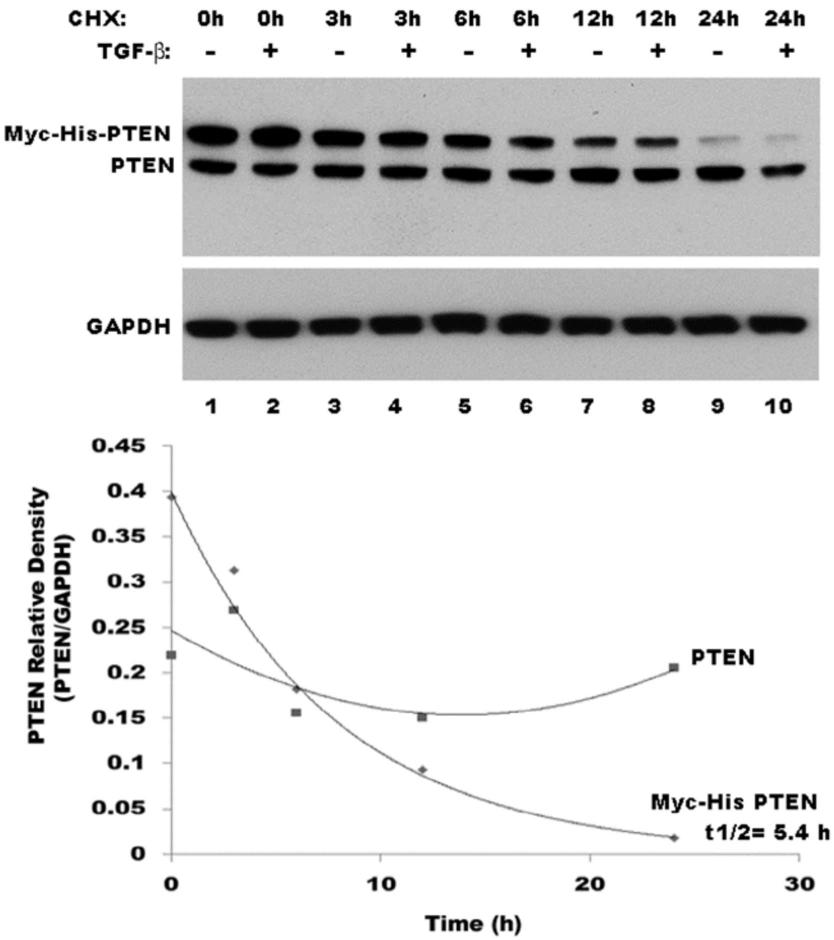

Supplemental Figure 4: Endogenous PTEN turnover is slower than Myc-His tagged PTEN**.** A) After 24h transfection of BUMPT cells with Myc-His-PTEN expression vector, cells were treated with cycloheximide (CHX, 10μM) alone or in combination with TGF-β (2ng/ml) and harvested at 0, 3, 6, 12, 24, h. Cell lysates were separated on SDS-PAGE, blotted and probed with PTEN and GAPDH antibodies. B) PTEN relative density was plotted against time after CHX treatment of BUMPT cells expressing Myc-His-PTEN.

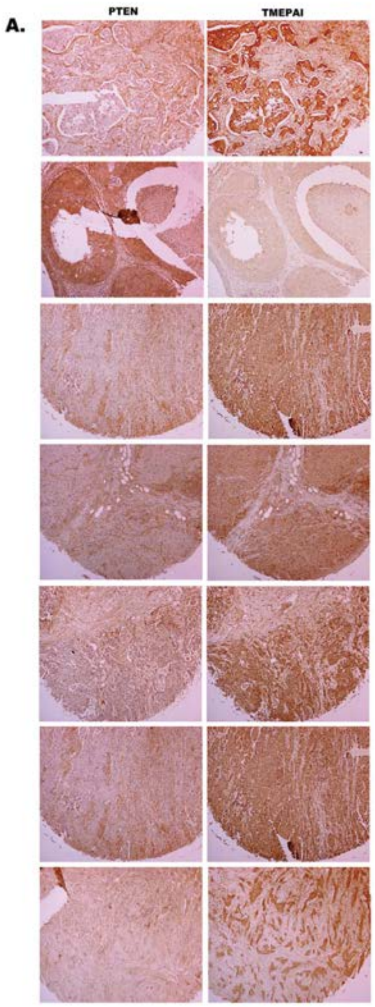

Supplemental Figure 5: Relative expression of TMEPAI and PTEN in TNBC patients measured by IHC. Immunohistochemistry was performed on human breast tissue arrays by staining for TMEPAI and PTEN antigens as mentioned in Materials and Methods section. A few comparisons are shown here. Staining was defined as positive for significant cytoplasmic immunoreactivity for PTEN and TMEPAI in cancer cells and was scored as: 0, 0-10% of positive cells; 1, 10–25%; 2, 25–50%; 3, 50-75% and 4, > 75%. Intensity was scored as 1, weak; 2, moderate and 3, strong. Final scores were obtained by multiplying the percentage of positive cells (P) by the intensity (I).
